# Unintended consequences of machine learning in medicine?

**DOI:** 10.12688/f1000research.12693.1

**Published:** 2017-09-19

**Authors:** Laura McDonald, Sreeram V. Ramagopalan, Andrew P. Cox, Mustafa Oguz

**Affiliations:** 1 Centre for Observational Research and Data Sciences, Bristol-Myers Squibb, Uxbridge, UB8 1DH, UK; 2 Evidera, London, W6 8DL, UK

**Keywords:** machine learning, healthcare, medicine, artificial intelligence

## Abstract

Machine learning (ML) has the potential to significantly aid medical practice. However, a recent article highlighted some negative consequences that may arise from using ML decision support in medicine. We argue here that whilst the concerns raised by the authors may be appropriate, they are not specific to ML, and thus the article may lead to an adverse perception about this technique in particular. Whilst ML is not without its limitations like any methodology, a balanced view is needed in order to not hamper its use in potentially enabling better patient care.

There is significant interest in the use of machine learning (ML) in medicine. ML techniques can ‘learn’ from the vast amount of healthcare data currently available, in order to assist clinical decision making. However, a recent article ^1^ highlighted a number of consequences that may occur with increased ML use in healthcare, including physician deskilling, and that the approach is a ‘black box’ and unable to use contextual information during analysis.

Whilst we agree that Cabitza *et al*’s concerns are justified ^1^, we believe that a more balanced discussion could have been provided with regards to ML-based decision support systems (ML-DSS). As it stands, an impression is given that ML is flawed, rather than the issue being the way in which it is applied. The concerns raised are generally applicable to many analytical approaches, and reflect poor study design and/or a lack of analytical rigour than the particular technique being used.

The authors cite two examples to claim that ML-DSS could potentially reduce physician diagnostic accuracy. The mammogram example ^2^ shows reduction in sensitivity for *6* of the most discriminating of 50 radiologists. However, the mammogram ML-DSS referred to is old ^2^, and it is not clear how the underlying model was trained and evaluated. The model may perform well for some types of cancer, but not as well for others as a result of the training data. Indeed updates have been shown to increase detection sensitivity ^3^. ML models can be refined by providing more data and results need to be critically appraised in this context. Additionally, no mention is made of the possible benefits of ML-DSS for less experienced staff. In the mammogram example, an improvement in sensitivity for 44 out of 50 ** radiologists was seen for easier to detect cancers. There was also an increased overall diagnostic accuracy when using ML-DSS in the electrocardiogram study ^4^. Accuracy loss for experienced readers when using ML-DSS is valid, but more reflective of training needed and not an outcome specific to ML-DSS. A knowledgeable doctor may have no need for an ML-DSS, but the tool could greatly assist less experienced staff.

Cabitza *et al.* also argue that the confounding caused by asthma in the outcome of patients with pneumonia would have not been observed in a neural network model. There are, however, methods to obtain the feature importance and the direction of the relationship between predictor variables and outcome in neural networks ^5^. Further, some ML approaches, such as random forest, are more transparent than others and ML can easily be coupled with clinical expertise to develop risk models that have their benefits over traditional statistical modelling ^6^.

The issues highlighted by Cabitza *et al.* are more concerned with the studies themselves rather than an intrinsic flaw in ML methodology. To fully leverage ML or any other approach, users must have a good understanding of the caveats. In summary, we agree that ML-based approaches are not without their limitations, but the growing application of ML in healthcare has the potential to significantly aid physicians, especially in increasingly resource constrained environments. Informed, appropriate use of ML-DSS could, therefore, enable better patient care.
