# Supplementary material for: Unintended consequences of machine learning in medicine?
Source: F1000Res. 2017 Sep 19;6:1707. [Version 1] doi: 10.12688/f1000research.12693.1 (PMC5701440; doi:10.12688/f1000research.12693.1)
Supplement: Peer review history

# Peer review: Referee response for version 1

**DOI:** 10.5256/f1000research.13746.r27518

**Published:** 2017-11-23

**Authors:** Hugo Schnack, Zimbo Boudewijns

**Affiliations:** 1 Department of Psychiatry, Brain Center Rudolf Magnus, University Medical Center Utrecht, Utrecht, Netherlands

Machine learning (ML) methods are currently being applied in a wide range of fields. Theoretically, the ability to extract meaningful relations from large datasets holds a great promise for health care and could potentially offer new, unexpected insights into disease and recovery.

 However, more critical voices have emerged warning of potential issues surrounding the use of ML and a number of points were addressed in the original paper by Cabitza *et al*. The authors of the current F1000 paper (McDonald *et al*) indicate they consider the issues raised as justified but call for a more balanced discussion regarding ML use in medicine. Indeed, Cabitza *et al*’s view is mainly negative. McDonald and colleagues correctly argue that it is often bad study design that leads to unreliable or unwanted outcomes and not the technique that is used. While a valid point, given how common bad study design or misuse of more common statistical techniques is, it is not unlikely that such issues will also arise from use of ML techniques in health care. Given the far reaching implications of unreliable AI-based decision support systems in health care careful scrutiny of the techniques is necessary.

 In their discussion of the reduced sensitivity of radiologists evaluating mammograms, McDonald and colleagues bring to the defense of ML techniques that the model that led to unwanted outcomes was old and that it might work better or worse depending on which type of cancer is being studied. In addition they state that it was not clear how the model was trained and evaluated and that having appropriate feedback loops in place can improve the accuracy of a model considerably. These are obviously important points that should always be taken into account both when evaluating a specific model as well as for the evaluation of the use of ML in medicine in general. The quality of the algorithm but also the quality of available data will ultimately decide how useful a ML approach is for a given medical problem. This only goes to show that ML models should be applied with care and does not negate the original concerns put forward by Cabitza *et al.*

 McDonald and colleagues also propose the use of more transparent ML techniques to prevent potential confounding variables that would not show in a black-box model. However, the predictive power of ML models often increases with their complexity, making transparency either very difficult or even impossible to obtain. As stated by the authors, users of ML models should have a good understanding of the limitations of the techniques. More often than not, ML models will be implemented in a collaboration between technical experts without medical knowledge and medical experts with limited technical expertise. Ensuring an optimal level of transparency of the models combined with the right amount of clinical expertise is therefore vital and while the authors mention this, there are no specific recommendations proposed to actually achieve this. With the increased application of ML models in health care there is a need for guidelines on how to optimally make use of the most technically advanced techniques while making sure bias in the data and confounding variables are accounted for. One recommendation the authors could have made is the need to bring the two worlds (medical and ML-technical) together, bridging the gap by educating people to become professionals with a (bio)medical *and* technical expertise, as well as training medical doctors in critically working with computerized predictions.

 Another point that could have been addressed is how to deal with the differences in experience between medical doctors. Superior performance of the most-experienced doctors could be used to improve the computerized models, while less-experienced doctors may likely gain expertise by reviewing the computerized predictions –provided the ML tool allows for a clear interpretation *how* it did come to its diagnosis. This property of ML algorithms should be further explored and developed, because it can and, in fact, should lead to further insight in the underlying mechanisms of diseases.

We have read this submission. We believe that we have an appropriate level of expertise to confirm that it is of an acceptable scientific standard.

# Peer review: Referee response for version 1

**DOI:** 10.5256/f1000research.13746.r26485

**Published:** 2017-10-30

**Authors:** Spiros Denaxas, Arturo Gonzalez-Izquierdo, Maria Pikoula

**Affiliations:** 1 Institute of Health Informatics, University College London, London, UK

The publication of this letter is both important and timely given the increased interest that statistical learning approaches applied to healthcare data are receiving. 

 The original article emphasized a negative perception of potential adverse consequences of machine learning (ML). It did not fully highlight the current benefits of using large amount of information for clinical decision making and the potential for methodological improvement with regards to statistical learning approaches. The very field of ML is rapidly evolving as illustrated by the rapid growth of deep learning over the past years.

 Minor comments

- Content could potentially be enhanced with a discussion on the notion that, precisely due to the outlined potential misuses and consequences, a systematic and strategic use of ML approaches must be developed and used to facilitate the robust application of such methods to healthcare data.
- The authors of the letter rightly point out that ML has similar advantages and drawbacks as any other analytical approach applied in a clinical setting. However, they fail to explain how clinician deskilling, which can be a real consequence of automation, could be averted or indeed whether it is an acceptable outcome, given overall positive effects. This is separate issue to that of algorithm predictive accuracy.
- "Accuracy loss for experienced readers when using ML-DSS is valid, but more reflective of training needed” - Is this training to improve interpretation of ML-DSS output in cases where it hinders correct reading? This could be relevant in the context of a study, however, it leaves open the possibility that in busy clinical settings, readers would be more likely to rely on computer aided detection.
- The authors could also highlight the potential for upskilling clinicians in the understanding of ML methods, which can enhance their interpretation of DSS and other data-driven processes. Clinicians could be invaluable in spotting when algorithms go wrong, even (and perhaps especially) for cases where they’ve been shown to overall outperform humans.
- Finally, the authors would make their case stronger by citing examples that demonstrate "proof of clinically important improvements in relevant outcomes compared with usual care, along with the satisfaction of patients and physicians”, as a concrete counter-balance to the negative or inconclusive examples of the original article.

We have read this submission. We believe that we have an appropriate level of expertise to confirm that it is of an acceptable scientific standard.
